# Exploring post-COVID-19 health effects and features with advanced machine learning techniques

**DOI:** 10.1038/s41598-024-60504-w

**Published:** 2024-04-30

**Authors:** Muhammad Nazrul Islam, Md Shofiqul Islam, Nahid Hasan Shourav, Iftiaqur Rahman, Faiz Al Faisal, Md Motaharul Islam, Iqbal H. Sarker

**Affiliations:** 1 Department of Computer Science and Engineering, Military Institute of Science and Technology, Mirpur Cantonment, Dhaka, 1216 Bangladesh; 2 Department of Computer Science and Engineering, Green University of Bangladesh, Dhaka, Bangladesh; 3 Department of Computer Science and Engineering, United International University, Dhaka, 1212 Bangladesh; 4 School of Science, Edith Cowan University, Perth, WA 6027 Australia

**Keywords:** COVID-19, Pandemic, Machine learning, Statistical analysis, Chi-square, Pearson’s coefficient, Computational biology and bioinformatics, Diseases, Health care, Medical research

## Abstract

COVID-19 is an infectious respiratory disease that has had a significant impact, resulting in a range of outcomes including recovery, continued health issues, and the loss of life. Among those who have recovered, many experience negative health effects, particularly influenced by demographic factors such as gender and age, as well as physiological and neurological factors like sleep patterns, emotional states, anxiety, and memory. This research aims to explore various health factors affecting different demographic profiles and establish significant correlations among physiological and neurological factors in the post-COVID-19 state. To achieve these objectives, we have identified the post-COVID-19 health factors and based on these factors survey data were collected from COVID-recovered patients in Bangladesh. Employing diverse machine learning algorithms, we utilised the best prediction model for post-COVID-19 factors. Initial findings from statistical analysis were further validated using Chi-square to demonstrate significant relationships among these elements. Additionally, Pearson’s coefficient was utilized to indicate positive or negative associations among various physiological and neurological factors in the post-COVID-19 state. Finally, we determined the most effective machine learning model and identified key features using analytical methods such as the Gini Index, Feature Coefficients, Information Gain, and SHAP Value Assessment. And found that the Decision Tree model excelled in identifying crucial features while predicting the extent of post-COVID-19 impact.

## Introduction

It is 2022-2023, and with the blessing of medical science, after the disastrous era of COVID-19, the world is finally seemingly healing from its wounds. But its deep-rooted adversities are still haunting the lives of the affected ones by the post-COVID trauma^1–3^. After a year of recovery, patients still find it challenging to return to everyday life. Many physical and Neurological factors indicate that vulnerabilities such as depression, anxiety, weakness, sleeplessness, etc., have increased alarmingly. Looking at the same person before and after their fight with COVID-19, it becomes clear of post-COVID trauma among them. COVID-19 has physical and neurological effects on our bodies^1^. And these types of factors are also interrelated with each other. For example, energy is significantly related to the sleeplessness of the patient.

Today’s physical and mental problems deeply connect with the patient’s previous COVID infection history^4^. These patients tend to be in mental traumas, neurological disorders, etc^5^. Research has also shown that COVID-19-recovered patients have common memory complaints and suffer from cognitive impairment, seizures, etc^5,6^. Thus it is important to explore whether any health problem in today’s era has any connection with the patient’s previous COVID history^7^. There has been much research on this phenomenon with modern approaches like statistical analysis and Machine Learning (ML) algorithms,^2,8,9^. Thus there comes the urgency for a comprehensive study with the help of statistics and ML models to evaluate the interrelation between before and after COVID health complications. Moreover, ML models may explore the interrelation between the COVID factors and how one factor can influence many others. Such findings can also be strongly supported by statistical analysis of the elements.

For example, machine learning analysis of Post-COVID-19 impact on medical staff and doctor productivity^10^ as well as the adverse effects and nonmedical use during the Pre- and Post-COVID-19 outbreak^11^; interpreting policy effects on air pollution during the COVID-19 lockdown in London with Explainable Machine Learning^12^; analyzing the impact of COVID-19 in KSA based on Arabic Tweets using Deep Learning^13^; understand the factors associated with mortality in COVID-19 hospitalized patients using ML^14^; assessing risks in SME supply chains due to Covid-19 disruptions^15^; Analyzing Spain’s social mood evolution during COVID-19 Vaccination based on Tweets using ML^16^; assessing the influence of COVID-19 on human personality^17^ and the effects on electricity consumption in distribution networks using ML^18^; evaluating COVID-19 characteristics and risk factors using the Bayesian Machine Learning and Markov Chain Monte Carlo Techniques^19^; analyzing factors influencing commercial crime calls using SHAP^20^ and the effects for COVID-19 patients with severe hypoxemia using Causal Bayesian ML^21^. Assessing the COVID-19’s psychological consequences using Deep Learnings^22^ as well as the Post-COVID-19 Recovery in urban area using Spatial and Deep Learning^23^.

Similarly, different models like Pearson’s coefficient and chi-square values determine how strongly they correlate. For example, the Mediating Influence of Resilience on Academic Stress, COVID-19 Anxiety, and Quality of Life in Nursing Students^24^.

Therefore, the primary objective of this research is to reveal various health issues related to post-COVID; secondly, to explore how much the revealed health factors have been impacted in post-COVID-19 individuals and how these factors are associated/correlated with each other; finally, to find the best-performed ML models for predicting the degree of impact of these health factors on post-COVID-19 individuals having different demographic profiles.

Our paper’s organization is as follows: In the opening section, we provide an introduction and delve into a literature review, with a particular emphasis on identifying the most significant features following the impact of COVID-19. The subsequent section offers a comprehensive view of our methodology and a presentation from an algorithmic standpoint. Moving to the third section, we unveil the results and engage in pertinent discussions. Finally, the fifth section serves as the conclusion of the paper and provides recommendations.

## Literature review

COVID-19 has had a major impact on humanity, as seen by the millions of verified cases and fatalities documented globally. Health, the economy, and interpersonal relationships are just a few areas in which the pandemic has significantly influenced. Many studies have been done in reaction to the epidemic to learn more about the virus, how it spreads, and potential cures and vaccinations. Scientists and healthcare experts are working nonstop to lessen the pandemic’s consequences and create successful long-term management plans. Shanbehzadeh et al.^1^ found some physical and mental issues in COVID-19 survivors with follow-up intervals of up to 3 months after COVID-19. The most frequent physical health issues were tiredness, pain, arthralgia, decreased physical capacity, reductions in physical role functioning, routine care, and daily activities. Anxiety, depression, and post-traumatic stress disorder were the three most prevalent mental health issues. Female patients and those admitted to critical care reported higher exhaustion, discomfort, anxiety, and sadness levels. Up to three months after COVID-19, overall, a lower quality of life was noticed. Matsumoto et al.^2^ work, it was found that 37.0% of the 763 participants, the 135 COVID-19 survivors had COVID-19-related aftereffects. First, the findings of the Mann Whitney U test with Bonferroni correction revealed that the SARS-CoV-2-infected group with post-COVID conditions had substantially higher scores on all clinical symptom measures than the non-infected group and those without one (P .05). The Chi-squared test findings showed that there was a significant difference in the incidence rates of clinically relevant mental symptoms among each group (P.001). Ultimately, the multivariate logistic model’s findings showed that participants with post-COVID disorders had a 2.44-3.48 times greater likelihood of experiencing more severe clinical symptoms. Additionally, Ahmed et al.^3^ showed that 16 individuals (8.8%) out of 182 had no sleep or mental health issues. 118 individuals (64.8%) reported having trouble sleeping, and 52 participants (28.6%) showed signs of probable PTSD. Somatization (41.8%) had the largest symptomatology percentage, followed by anxiety (28%), anger-hostility (15.9%), phobic anxiety (24.2%), obsessive-compulsive (19.8%), interpersonal sensitivity (0.5%), depression (11.5%), paranoid ideation (10.4%), and psychoticism (17.6%).

García-Sánchez et al.^25^ discussed that attention abilities had a widespread influence, both as the only impacted domain (19% of single-domain impairment) and in combination with lowered performance in organizational processes, learning, and long-term memory. These prominent executive and attentional impairments were essentially independent of clinical elements like hospitalization, the severity of the illness, biomarkers, or emotional assessments. For the first time, Benedetti et al.^4^ , explored the post-acute COVID-19 syndrome, inflammatory markers during acute COVID, brain regional GM volumes, DTI assessments of WM microstructure, and resting-state functional connectivity. The significant findings are that post-traumatic symptoms and decreasing GM volumes in the ACC and bilateral insular cortex correlate with WM microstructure and that depressed psychopathology correlates with decreasing GM volumes in the ACC. Moreover, resting-state FC was linked to inflammation and psychopathology, supporting the idea that the structural effect impacts brain function. Tarsitani et al.^6^ saw concerningly high rates of PTSD and subthreshold PTSD in hospitalized COVID-19 patients. The proven risk factors for PTSD include female sex and pre-existing mental illnesses. After patients are discharged from the hospital, clinicians treating COVID-19 patients should think about checking for PTSD during follow-up examinations. Besides, Ahmed et al.^5^ examined and found that 19.2% of COVID patients had memory difficulties in the study. He also discovered that steroids and antibiotics were linked to memory impairment among the treatment modalities, according to individual predictor analyses. According to multiple logistic regression, those who recovered from COVID-19 within six to twelve months were more likely to suffer memory problems. Although there was no correlation between age, sex, oxygen demand, or hospitalization and memory problems, rural inhabitants had more serious memory complaints than urban residents.

Moreover, Sher^26^ found that psychiatric, neurological, and physical disease symptoms are likely to exacerbate suicidal ideation and behavior in this patient population, as are brain inflammation and post-COVID syndrome symptoms. Without post-COVID syndrome, COVID-19 survivors may potentially have a higher risk of suicide. More proof is identified by Pistarini et al.^27^ described patients with cognitive abnormalities who were treated in COVID and post-COVID functional rehabilitation programs. According to the MoCA examination, specifically, 75% of COVID patients and 70% of post-COVID patients showed cognitive abnormalities. These findings demonstrate the severity and protracted nature of the neurological and mental effects that can result from COVID-19 infection.

To sum up, no review study explicitly focused on exploring all possible health issues or factors in post-COVID-19 patients. Moreover, limited research has been conducted to evaluate the impact of the factors after COVID-19 recovery. Whether or not any relationship exists among these health factors has fallen into the research gap. Besides, the application of ML models to detect post-COVID-19 issues needs to be explored in the research area.

Here, we have listed the main contribution of the research:

1. Explore health complications related to post-COVID-19 by identifying 17 significant Physiological and Neurological health factors.
2. Examine the independent influence of each factor and their interconnections rigorously.
3. Select the most important feature named Anxiety from the outcome of the best-performed four ML models (with feature ranking and comparative analysis); and the Decision Tree algorithm demonstrated the highest accuracy in predicting post-anxiety levels.

## Methodology overview

**Figure 1:**
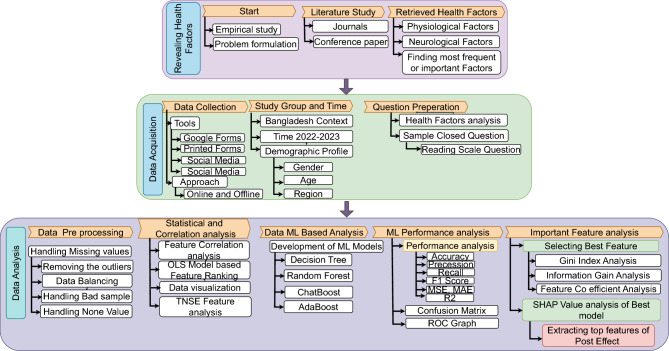
Methodological overview.

The research methodology is divided into three phases as shown in Fig. 1. Firstly, we explored the health factors by reviewing the related literature. Secondly, necessary data were collected from a study group, i.e., post-COVID individuals. Finally, data analysis was performed through statistical and ML-based approaches. Extracting top features of post effect of best-performed ML models.

### Exploration of the health factors

We have reviewed various research articles published in 2022 and 2023 to explore the health factors. Our aspect is the post-COVID scenario, so we stuck to research articles limited to this genre. At the same time, the search was performed in scholarly databases like IEEE Explore, Google Scholar, ACM, Digital Libraries, ResearchGate, etc. As an outcome, 17 post-COVID health complications or factors were revealed, such as stress disorders, cognitive impairment, impulsiveness, etc. The indicated factors were categorized into two major categories: Physiological Factors(Chest pain, sleeplessness, fainting) and Neurological Factors(Anxiety, depression, confidence).

### Data acquisition

1. *Preparing questionnaire:* A questionnaire with a total of 13 questions was prepared by considering all (17) revealed factors, each having questions related to the condition before COVID-19 and another related to the health condition after COVID-19. The Rating Scale for the target class in numeric value as 5, 4, 3, 2, 1 for Strongly Agree, Agree, Neutral, Disagree, and Strongly Disagree respectively.
2. *Data features:* In our data set we have used 13 input features named as Features list: $''gender''$, ”age”, ”education”, $''heart_{disease}''$, ”diabetis”, $''other_{disease}''$, “smoking”, $''blood_{pressure}''$, ”weight”, $''work_{type}''$, “married”, $''vaccination_{status}''$, $''vaccination_dose_status''$. The total respondents were composed of 600 males and 400 females. All the respondents were vaccinated, and their average age was from 10 to 70 years old. The data has other input samples as : Education category: Higher, mid, and low study, Heart disease category: Yes or No, Diabetes category: Yes or No, Other disease category: Ye or No, Smoking category: Yes, Never, Partial, Blood pressure category: Low, Mid and High. Weight category: High, Mid and Low, Working type: Pvt Job, Self Employed, Govt job and Unemployed, Married category: Yes or No. Vaccination status: Yes or No. Number of vaccination doses taken: One, One with Two, and One, Two with Booster.
3. *Study group*: The survey questionnaire was distributed among people in Bangladesh of different age groups, genders, etc. 1000 people with different demographic profiles participated who all suffered from COVID-19.
4. *Data collection approach:* The questionnaire set was primarily distributed among the students and faculty members of the authors’ institute via email or Physically. The questionnaire was also distributed following the online distribution methods. Respondents were given two weeks to respond. Moreover, as an Offline approach, we hosted temporary places for volunteer participation and set provisions for gifts for kind participation. Finally, a total of 1000 responses were collected. The whole data collection process was carried out from July 2022–August 2023.

### Data validation

Following data collection, we conducted data validation through the expertise of two distinguished medical professionals from renowned institutions in Bangladesh. These two doctors put forth their utmost diligence in labeling the data.

### Institutional approval and ethical confirmations

1. We confirm that all methods were carried out in accordance with the relevant ethical guidelines and regulations by the Research and Development wings of the Military Institute of Science and Technology (MIST), Dhaka-1216.
2. We confirm that all experimental protocols were approved by the Research and Development wings of the Military Institute of Science and Technology (MIST), Dhaka-1216. This research and its data collection and analysis confirm that all the informed consent was obtained from all subjects and/or their legal guardian(s).

### Data sample

Data samples are illustrated in the Table 1, only 10 samples are given in the table for the target class Anxiety after COVID-19. Other target classes (Post covid effect) are not shown in the table.

**Table 1:** Data sample for the target class: anxiety after.

Gender	Age	Education	Heart disease	Diabetes	Other disease	Smoking	Blood pressure	Weght	Work type	Married	Vaccination status	Vaccination dose status	Anxiety before	Anxiety after
Female	10-20 years	Higher study	Yes	Yes	Yes	Yes	Low	High	Pvt Job	Yes	Yes	1 and 2 Dose	Neutral	Neutral
Female	41-50 years	Higher study	Yes	Yes	Yes	Never	Low	High	Pvt Job	Yes	Yes	1, 2 and Booster Dose	Agree	Agree
Male	10-20 years	Higher study	Yes	Yes	Yes	Never	Low	High	Pvt Job	Yes	Yes	1 and 2 Dose	Agree	Agree
Female	31-40 years	Higher study	Yes	Yes	Yes	Never	Low	High	Pvt Job	Yes	Yes	1, 2 and Booster Dose	Agree	Strongly agree
Female	21-30 years	Higher study	Yes	Yes	Yes	Never	Low	High	Pvt Job	Yes	Yes	1, 2 and Booster Dose	Neutral	Neutral
Female	31-40 years	Higher study	Yes	Yes	Yes	Never	Low	High	Pvt Job	Yes	Yes	1, 2 and Booster Dose	Neutral	Agree
Male	31-40 years	Higher study	Yes	Yes	Yes	Never	Low	High	Pvt_Job	Yes	Yes	1, 2 and Booster Dose	Disagree	Agree
Male	21-30 years	Higher study	No	Yes	Yes	Never	Low	High	Pvt Job	Yes	Yes	1, 2 and Booster Dose	Disagree	Disagree
Female	31-40 years	Higher study	No	Yes	Yes	Never	Low	High	Self employed	Yes	Yes	1, 2 and Booster Dose	Agree	Strongly agree
Male	21-30 years	Higher study	No	Yes	Yes	Yes	Low	High	Self employed	Yes	Yes	1, 2 and Booster Dose	Neutral	Disagree

### Data analysis

#### Statistical analysis

In this step, we statistically analyzed every factor for the before-COVID-19 and after-COVID-19 state. For example, the symptom of Anxiety is investigated for both conditions (before their COVID-19 infection and after the infection).

#### ML based analysis

After the statistical analysis, the data were trained through various traditional ML models like Decision trees, Random Forest, and Ensemble ML Models such as Adaptive boosting, Gradient boosting, and Extreme gradient boosting.

#### Evaluation of ML models

Various parameters like Accuracy, Precision, Recall, and F1 score measured the performance of the ML models. The Confusion Matrix was implemented to judge the accuracy along with ROC analysis.

### **Study outcomes**

To achieve our objectives, we conducted an in-depth analysis of the major health complications associated with COVID-19. A comprehensive overview of our findings is presented in Fig. 1. Our research identified 17 significant health factors, categorized as Physiological and Neurological, which played a pivotal role in our study. Using these factors as a foundation, we conducted surveys with individuals who had recovered from COVID to assess their conditions both before and after their illness. We rigorously subjected this survey data to statistical analysis, unveiling how each of these 17 factors independently influences patients and exploring their interconnections. This marks the accomplishment of our second objective. Subsequently, we proceeded to identify the most effective predictive models for determining the extent of influence exerted by these health factors. Notably, the Decision Tree algorithm exhibited the highest accuracy in predicting anxiety levels, which serves as our ultimate objective. In our final stage, we identified the key features in the post-effect of the best-performing machine learning model. We employed a variety of methods, including feature importance analysis, Gini index, information gain, feature importance permutation, and SHAP value analysis, to uncover these essential insights of important features of post-COVID-19 effects. The primary outcomes of our study are:

1. Our research focused on analyzing major health complications related to COVID-19 by identifying 17 significant health factors categorized as Physiological and Neurological.
2. We conducted surveys with recovered COVID-19 patients to assess the impact of these factors on their health before and after their illness.
3. We rigorously analyzed the survey data to examining the independent influence of each factor and their interconnections.
4. We chose the most important feature named Anxiety from the outcome of survey study frequency. Among four ML models, the Decision Tree algorithm demonstrated the highest accuracy in predicting anxiety levels.
5. In our final stage, we identified key features in the post-effects of the best-performing machine learning model through various methods, providing valuable insights into post-COVID-19 effects.

## Revealing the health factors due to COVID-19

In the last two and a half years, the COVID-19 pandemic has drastically affected millions worldwide. The impact hammers on physical and mental health problems in the post-COVID-19 state^1^. This phenomenon raises the necessity to investigate the relationship between post-COVID conditions and mental health^2^. Primarily, the investigation shows that coronavirus has a long-term effect of post-COVID-19 disease on sleep and mental illness, which also opens the door to detecting possible relationships between the severity of COVID-19 at the onset and sleep and mental illness^3^. Coronavirus affects the brain by bypassing the blood-brain barrier (BBB) in blood or via monocytes which could reach brain tissue via circumventricular organs^7^. Importantly, research shows a prominent frequency of impaired performance across cognitive domains in post-COVID patients with subjective complaints^25^. At the same time, the discovery of inflammatory biomarkers in COVID-19 survivors has come into broad light through MRI samples and other means^4^. One out of five patients hospitalized for COVID-19 was diagnosed with PTSD or subthreshold PTSD at a 3-month follow-up^6^. Potential contributing factors cause post-COVID-19 patients to suffer from different memory complaints^5^. Moreover, some psychiatric issues like ’depression’ prevail in COVID recovery patients, which causes a 25 times greater risk for suicide than the general population^26^. A summary of data from last year about the impacts on physical, cognitive, and neurological health disorders in COVID-19 survivors suggests three crucial aspects to manage: nutritional status, neurological disorders, and physical health^28^. So, the impaired cognitive deficits and emotional distress among COVID-19 patients should be addressed by functional rehabilitation^27^. Side by side, a brief study is to be analyzed on post-COVID-19 pandemic era mental health issues, vulnerable populations, and risk factors, as well as recommending a universal approach for mental health care and services^29^. Physiological and Neurological factors have been examined, with 39% classified as Physiological and 61% as Neurological. Neurological factors influence the mind and are connected to a person’s mental and emotional state.^30^. Here anxiety is a major Neurological factor among post-COVID patients with a frequency rating of 8 as shown in Table 2. Anxiety is the most common mental illness in post-COVID^1^. Physiological factors deal with the functions of a living organism and its parts^30^. Fatigue is one of the most frequent alterations of post-COVID patients as shown in Table 2. Over the past three years, extensive research has explored physiological and neurological health complications in the aftermath of COVID-19. We reviewed 23 research articles using keywords like mental health, cognitive impairment, and post-COVID trauma. From these studies, we identified 17 health factors associated with COVID infection, including fatigue, forgetfulness, and anxiety. These factors were categorized into two groups: Physiological and Neurological. Notably, 39% are Physiological factors, while 61% are Neurological factors, impacting the mind and emotional well-being^30^. Here anxiety is a major neurological factor among post-COVID patients with a frequency rating of 8 as shown in the Table 2. Anxiety is the most common mental illness in post-COVID^1^. Physiological factors deal with the functions of a living organism and its parts^30^. Fatigue is one of the most frequent alterations of post-COVID patients Table 2.

In this way, all revealed health factors are listed in Table 2 along with references and frequency of presence in those references.

**Table 2:** Revealed health factors from literature summary.

Categorical health factors	Refs.	Frequency
Anxiety/obsessive-compulsive(OCD)/phobical-anxiety	^2–6,10,12^	8
Depression	^2,3,5,9–12^	7
Insomnia/poor sleep/ sleeping disorder	^2,3,5,6,12^	5
Fatigue/chronic fatigue/ Anti-vigilant condition	^2,3,6,9^	4
Impulsiveness	^5^	1
Fear	^3^	1
Dizziness	^3,6,9,11,12^	5
Drug addiction	^9,11–13^	4
Attentiveness/cognitive disorder/ lack of attention	^8,9,11–13^	5
Seizures	^1,19^	2
Forgetfulness/memory loss/ long-term memory deficits	^8,13^	2
Chest pain	^14^	1
Unhappiness	^15^	1
Anger	^16^	1
Confidence	^17^	1
Patience	^18^	1
Fainting	^20^	1
Energetic	^19^	1

Among the 17 factors we have divided them into two categories, as shown in Table 2;

1. *Physiological factors*: Physiological factors deal with the functions of a living organism and its parts^30^. For example, fatigue is one of the most frequent alterations of post-COVID patients in Table 2. There are 7 physiological factors identified among all post-COVID-19 factors in this study, as shown in Table 2.
2. *Neurological factors*: Neurological factors are the one that influences or affects the mind and are related to the mental and emotional state of a person^30^. For example, anxiety is the most common mental illness in post-COVID^1^. There are 10 neurological factors identified among all post-COVID-19 factors in this study, as shown in Table 2.

### Statistical analysis

**Figure 2:**
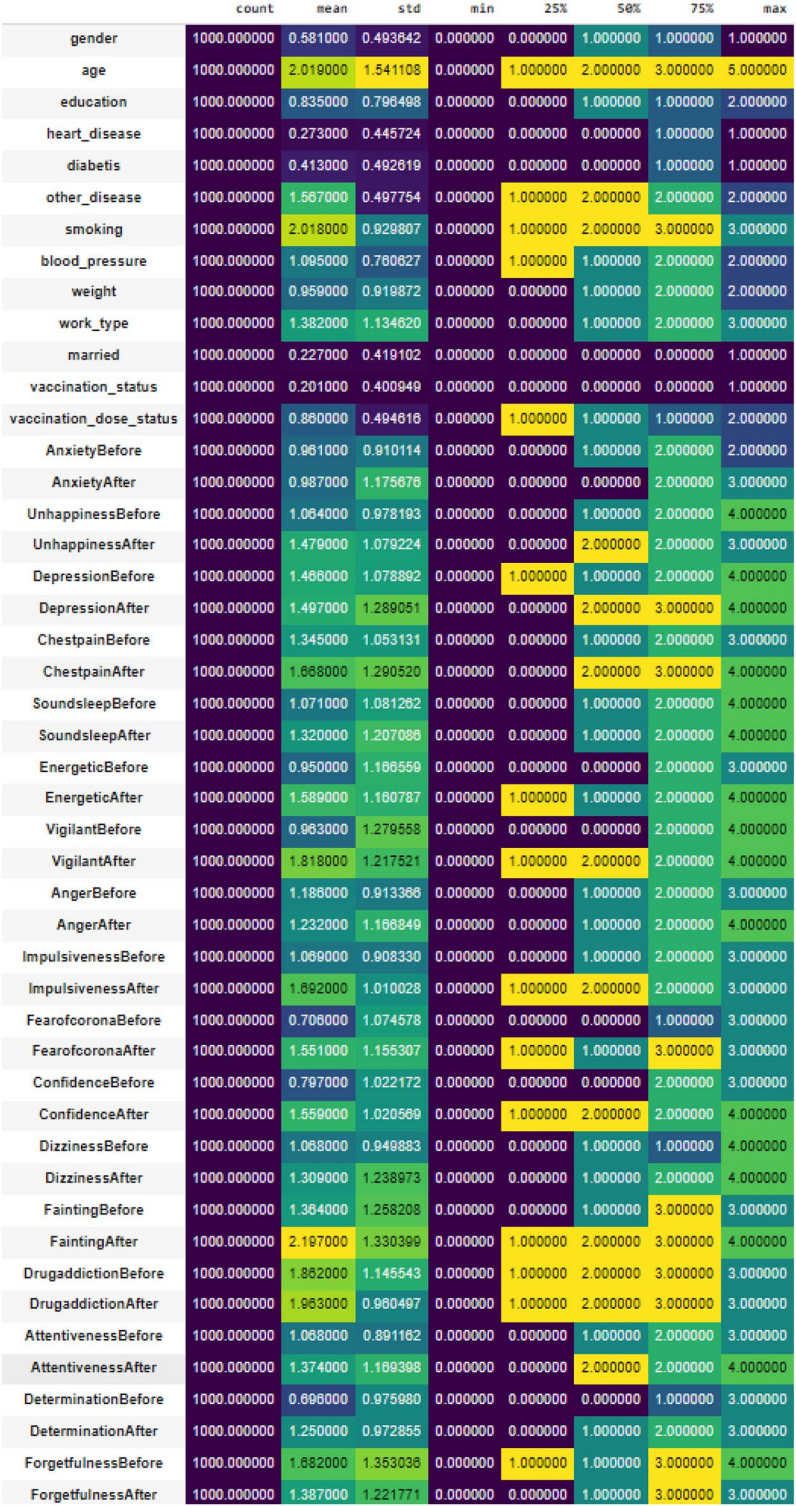
Statistical overview of data.

We have given a statistical overview of our data in Fig. 2 to make our data more understandable. Data statistics, such as count, min, max, mean, standard deviation, variance, and median, are essential for understanding a dataset. Count shows dataset size, min/max indicates its range, mean reflects central tendencies, standard deviation measures data spread, and variance quantifies overall variability. The median is a robust central measure. These stats form the foundation for data summary, with quartiles, percentiles, skewness, and kurtosis for deeper dataset analysis.

#### Feature correlation

**Figure 3:**
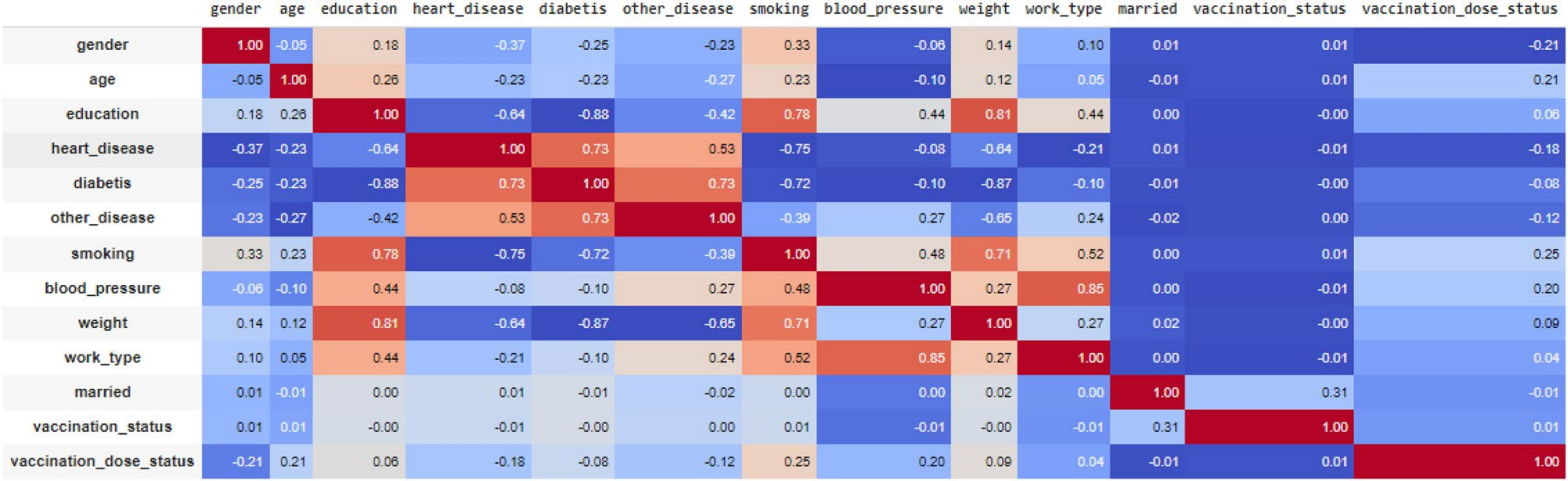
Pearson correlation value for all to all input features.

**Figure 4:**
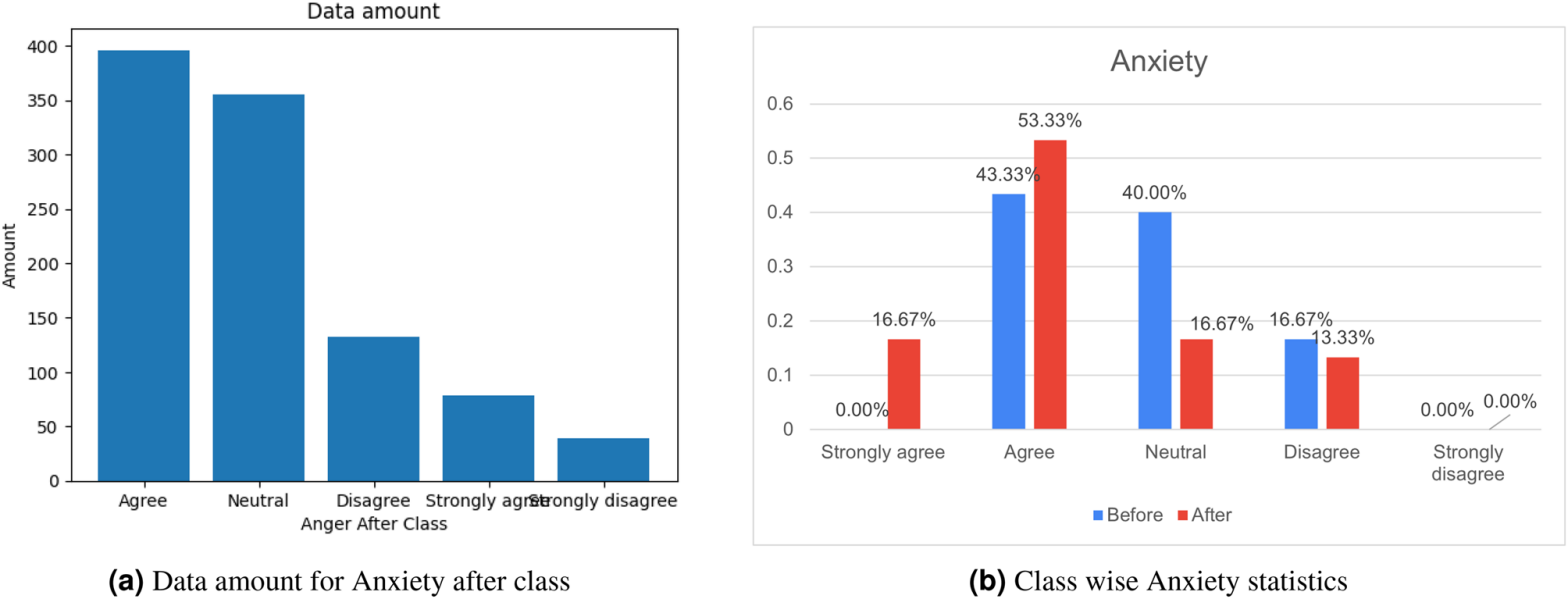
Overview of target class—anxiety.

**Figure 5:**
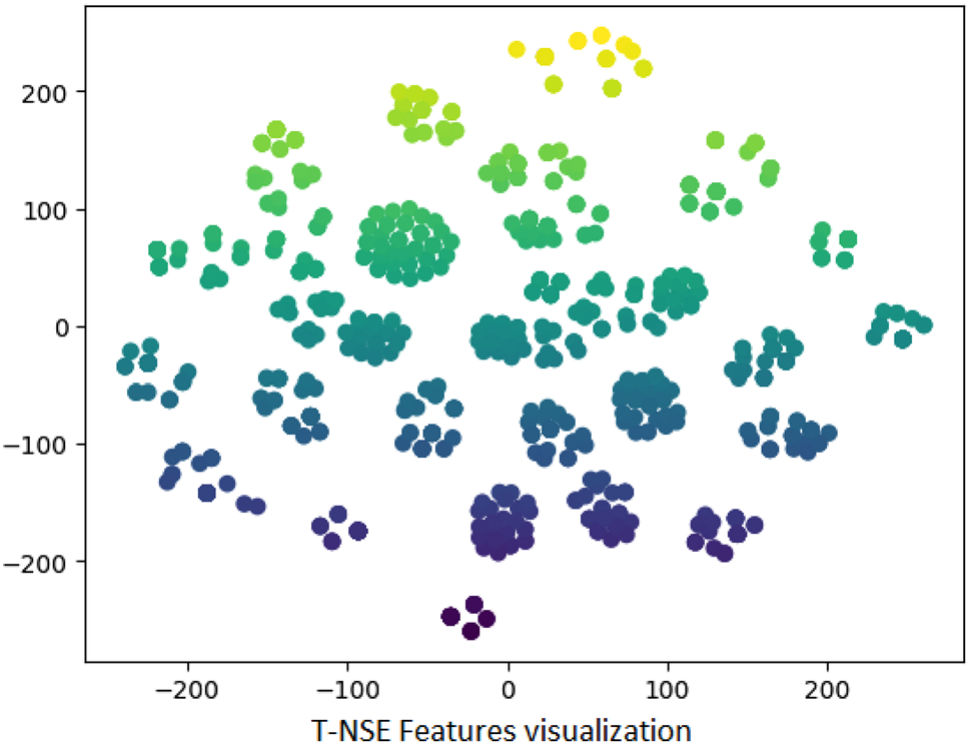
TNSE visualization of features for after anxiety.

Feature correlation in Figs. 3 and 4 gives a statistical measure that assesses the degree of association or relationship among features (variables) in our dataset. It quantifies how these features tend to vary together, providing insights into their dependencies. The advantages of this feature correlation (pearson) analysis in Fig. 4 (Full information is shown in Fig. 5) includes its utility in identifying redundant or highly informative features for best model performance, detection of multicollinearity in regression analysis, simplifying data exploration by revealing hidden patterns and relationships, aiding in model interpretability, and facilitating feature engineering by leveraging the knowledge of feature associations to create new informative variables. Pearson correlation, is a crucial data science tool. It quantifies the strength and direction of the linear relationship between two continuous variables, with values ranging from −1 to 1. This technique is widely employed in statistics and data analysis to uncover connections, patterns, and dependencies within complex datasets.

#### Evaluating significant association

The chi-square test is one of the methods to find out the association i.e. relationship among the categorical variables. The relationship can be significant or insignificant. The standard P-value is considered as 0.05 and any p-value having less than 0.05 is considered to have a significant association i.e. relationship among variables as shown in Fig. 3. In this research, the survey dataset has the responses i.e. level of impact on various physiological & neurological factors. These factors are considered categorical variables. The chi-square test is applyed on all factors and we got *P*-value for them which is shown in Fig. 3. In the Table 3, calculated p-values less than 0.05 are marked with Grey color. These values with corresponding Factors are analysed to possess significant relationships among them.

From the Fig. 3, we can see all comparing factors have an association between them, Some basic features association as follows: a. Chest Pain & Unhappiness b. Unhappiness & Forgetfulness c. Depression & vigilance d. Chest pain & confidence e. Confidence & vigilance f. Energy & confidence g. Sleep & attentiveness h. Attentiveness & vigilance i. Sleep & determination j. Determination & vigilance and k. Fear of COVID & energetic

#### Exploring positive and negative correlation

Pearson correlation coefficient is a unit measuring the strength of the linear relationship between two variables. This is represented as the ’r-value.’ ’R-value’ results in the range from −1 to 1. +1 represents the positive correlations(direct relationship), 0 shows no relationship & −1 represents the negative correlations(inverse relationship). In the research, the physiological & neurological factors of the dataset are depicted as variables. The Pearson correlation coefficient is calculated for all factors, and we got the R-value for them shown in Fig. 3. The R-values above 0.05 are considered for positive/direct relation between the factors. This means an increase in one factor may influence and increase the degree of another factor. R-values below 0(in the -ve range) are considered for Inverse relation between factors. This means a Decrease in one factor may influence and Decrease another factor. The Pearson correlation revealed a strong positive relationship between the two variables, with a correlation coefficient of 0.85, indicating a significant and direct association.

#### Feature ranking using regression model OLS

Feature importance analysis shown in Fig. 3 using the Ordinary Least Squares (OLS) regression model is a valuable technique in data analysis and predictive modeling. In this table, we renamed each feature name and labeled it from 1 to 13. In the context of feature importance, OLS can reveal the impact of each independent variable on the dependent variable. Larger coefficient values indicate stronger feature importance, while coefficients near zero suggest less relevance. This analysis aids in feature selection, helping us focus on the most influential variables for building predictive models or understanding the factors that drive specific outcomes in the data. Based the outcome shown in Table 3, the most important feature is 13(with a score of 1.5447) and the less important feature is 1(with a score -1.0443).

**Table 3:** Feature ranking based on OLS model outcome analysis.

OLS regression results						
Dep variable		y		R Squared		0.455
Model		OLS		Adj R squared		0.449
Method		Least squares		F-statistics		71.61
Prob (F Statistics)		1.4ee−236		Log likelihood		-1363.5
No of observation		1000		AIC		2753
DF Model		12		BIC		2817
Covariance type		Non Robust		Df Resudials		1000
Omnibus		31.703		Durbin Waston		2.412
Skew		0.389		Kustosis		2.661
Jarque-Bera		31.293		Prob JB		1.60e-07

Features	Coefficient	Standard error	t	p t	0.025	0.975
1	−1.0443	0.074	−14.090	0.00	−1.190	−0.899
2	−0.1990	0.026	−7.738	0.00	−0.249	−0.149
3	1.5447	0.164	9.430	0.00	1.223	1.866
4	−1.0476	0.123	−8.505	0.00	−1.289	−0.806
5	3.0705	0.334	9.183	0.00	2.414	3.727
6	−0.8009	0.137	−5.863	0.00	−1.069	−0.533
7	−0.5227	0.075	−7.004	0.00	−0.669	−0.376
8	−0.5026	0.104	−4.837	0.00	−0.707	−0.299
9	−0.2789	0.079	3.526	0.00	0.124	0.434
10	0.2906	0.065	4.443	0.00	0.162	0.419
11	−0.2716	0.342	−0.795	0.427	−0.942	0.399
12	−0.2716	0.342	−0.795	0.427	−0.942	0.399
13	0.5735	0.073	7.814	0.00	0.430	0.718

**Algorithm 1:**
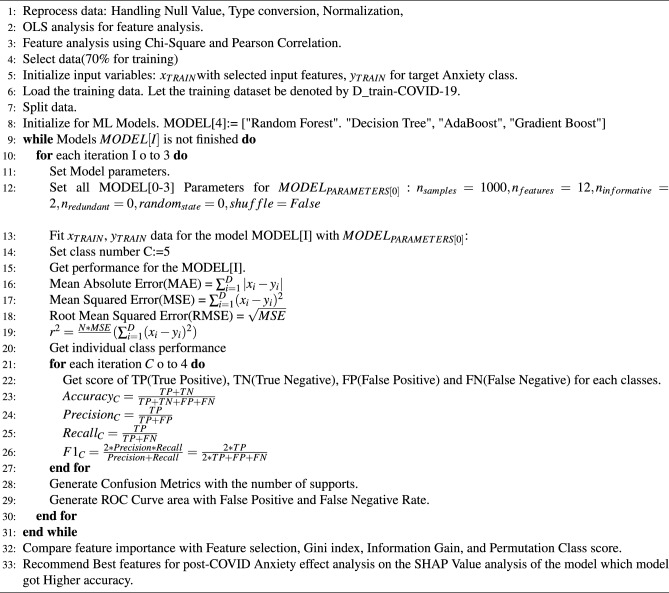
Training algorithm for anxiety analysis.

### Impact on post-COVID-19 health factors: before-to-after

Firstly, the compiled dataset is used for Statistical Analysis to explore whether any impact exists on the factors due to COVID-19 or not. The dataset possesses the info of both the Before and After conditions of the factors. The x-axis shows the categories/responses of people on how much each factor, like anger, depression, etc is affected. Y-Axis shows the percentage of how many persons are acknowledged in each category. In Fig. 4b, we present a comparative view of anxiety before and after COVID-19. The blue color represents the degree of impact for the factors before being affected by COVID-19. The red color represents the status after suffering from the disease.

#### Anxiety

Before COVID-19 state, no people strongly agreed on having Anxiety over their COVID issue, but the percentage jumped to 16.67% who strongly agreed after suffering from it. The graph follows the same pattern in the subsequent remarks. Comparing the before & after situations, it can be concluded that after suffering from COVID-19, a large number of people got the new problem whereas the people having previous Anxiety issues remained the same/more. In Fig. 4a, we present a complete view of anxiety amount before and after COVID-19.

#### Depression

It is such a factor that shows most of the patients are suffering from depression more after COVID. 23.33% and 36.67% patients either strongly agreed or agreed respectively on this matter. This figure has risen from 16.67% and 20.00% before COVID. While 36.67% disagreed on this matter before COVID the figure came down to only 10.00% after COVID. Depression, in human life, has increased after COVID-19

#### Unhappiness

On the factor of unhappiness, 33.33%, and 26.67% people agreed on their unhappy life before and after COVID respectively. However we see an almost inverse trend on the neutral point of view among the patients. Thus comparing the before & after situation, it can be visualized that after suffering from COVID-19, unhappiness has decreased among the patients.

#### Confidence

The degree of confidence before and after the COVID-19 era shows a drastic change in people’s mentality. Before COVID-19 state, 56.67% of people agreed on their degree of confidence but COVID had hit hard on their lifestyle shifting down to 20% confidence degree after COVID. The same trend was seen in the disagreement chart. Comparing the before & after situation, it can be concluded that after suffering from COVID-19, the majority of the people’s confidence in themselves was shattered.

#### Forgetfulness

Regarding forgetfulness, double the number of patients either agreed or strongly agreed that they forgot things now more after suffering from it. Thus, COVID has fatally affected the patients’ memory, resulting in curbing their brains.

#### Patience

Before suffering from COVID, about 60% people agreed that they were more patient in life, but the percentage abruptly dropped to half who decided to be after suffering from COVID. But none Strongly Disagreed in this regard, neither before nor after. Thus comparing the before & after situation, it can be visualized that after suffering from COVID-19, vigilance has decreased by almost half or beyond among the patients.

#### Energetic

Before the COVID-19 state, most people (56.67%) agreed about being more energetic, whereas the percentage increased in favor disagreement (36.67% disagree, 10% strongly disagree) in the post-COVID state. Comparing the before & after situations, it can be depicted that after suffering from COVID-19, people are becoming significantly less energetic.

#### Chest pain

Before COVID-19 state, no people strongly agreed about having chest pain, but the percentage jumped to 23.33% who strongly agreed after suffering from COVID. Comparing the before & after situations, it can be concluded that after suffering from COVID-19, a large number of people got the new problem, whereas the people having previous chest pain history remained the same/more.

#### Sleep

Before COVID-19 state, about 36.67% of people agreed that they experienced more sleep, but the percentage decreased to 33.33% who agreed after suffering from COVID. Comparing the before & after situations, it can be concluded that after suffering from COVID-19, experiencing sound sleep conditions shows a sight-decreasing tendency.

#### Anger

Before COVID-19 state, about 43% of people were NEUTRAL about their anger problem, whereas 40% people agreed about the problem. Comparing the before & after situations, it can be concluded that after suffering from COVID-19, most people agreed that their anger has increased.

#### Dizziness

Before the COVID-19 state, most people (50%) disagreed about having dizziness problems, but the percentage is rising in favor of strongly agree (16.67%) and agree (36.67) in the post-COVID state. Comparing the before & after situations, it can be concluded that after suffering from COVID-19, dizziness is slowly increasing among people after COVID.

#### Impulsiveness

Before the COVID-19 state, a few people (3.33%) strongly agreed that they had been impulsive, but the percentage increased to 20% who strongly agreed after suffering from COVID. Comparing the before & after situations, it can be concluded that after suffering from COVID-19, people show a sight-increasing impulsiveness tendency.

#### Vigilance

Before suffering from COVID, about 60% of people agreed that they were more vigilant, but the percentage abruptly fell to 16.67% who agreed after suffering from COVID. At the same time, disagreement degrees increased in the post-COVID situation. Comparing the before & after situations, it can be visualized that after suffering from COVID-19, vigilance has decreased dramatically among the patients.

## Determining correlation among health factors: factor-to-factor

The revealed health factors are analysed to check whether any significant or meaningful relationship exists between them.

### Evaluating relationship among health factors

The preprocessed dataset visualizes some important information. Explored information shows obvious relationship among the Health Factors. Bar-chart shown in Fig. 4b depicts the inherent relationship between two factors(like After Anxiety-to-before Anxiety). Various factors revealed a significant relationship. They are illustrated below :

#### Anxiety-to-energetic

About 53.33% of people agreed on Anxiety problems after suffering COVID-19 which is higher than the number of people (43.33%) who agreed before COVID-19. Again, 16.67% of people strongly agreed after COVID-19, whereas no person strongly agreed. Besides, 56.67% people agreed & 13.33% people strongly agreed that they were more energetic before COVID-19, whereas only 16.67% people agreed & 6.67% people strongly agreed on the issue after COVID-19. It can be seen that the amount of disagreement is higher, which is about 36.67% after the COVID-19 state, which means that patients got less energetic after COVID-19. Thus, Fig. 4b visualizes that Anxiety has increased among the patients. At the same time, they become less energetic after suffering from COVID-19.

#### Depression-to-vigilance

About 36.67% of people agreed on having depression after suffering from COVID-19, which is higher than the number of people (20%) who agreed before COVID-19. Again, 23.33% people strongly agreed after COVID-19, whereas 16.67% strongly agreed on the issue before COVID. Besides, 60% people agreed that they were more vigilant before COVID-19, whereas only 16.67% people decided after COVID-19. It can be seen that the amount of disagreement is more (Neutral, Disagree, Strongly disagree) after the COVID-19 state means that patients are becoming less vigilant after COVID-19.

Thus, the graph outlines that depression has increased among the patients. At the same time, they have become less vigilant after suffering from COVID-19.

#### Confidence-to-energetic

More than half of the people (56.67%) agreed on having more confidence before COVID-19, which is higher than the number of people (20%) who agreed after suffering from COVID-19. At the same time, 56.67% people agreed that they felt more energetic before COVID-19 but only few people (16.67%) agreed after COVID-19.

Thus, the graph shows that the Confidence degree has decreased abruptly among the patients with the sudden decrease in energy degree after suffering from COVID-19.

#### Chest pain-to-unhappiness

The graph shows that about 23.33% people strongly agreed that they got more or newly generated chest pain at post-COVID state. Besides, about 20% of people said strongly about their unhappy state after suffering from COVID.

Thus, the graph represents that a considerable amount of people have grown chest pain which causes an unhappy state of people higher than previous.

#### Confidence-to-chest pain

More than half of the people (56.67%) agreed on having more confidence before COVID-19 which is higher than the number of people (20%) who agreed after suffering from COVID-19. Besides, 23.33% of people strongly agreed that they grew more chest pain after COVID-19 whereas no person strongly agreed before COVID-19.

Thus, the graph shows that the Confidence degree has decreased among the patients. At the same time, there is a high tendency to gain chest pain after suffering from COVID-19.

#### Sound sleep-to-attentiveness

This graph shows that there is a slight decrease in patients’ sleep conditions before & after suffering from COVID-19. Similarly, the percentage of attentiveness also a little low situation in pre & post-COVID-19 situations.

Thus, the graph shows that the degree of sleep & attentiveness slightly decreased in post COVID state.

## Development of predictive models

In our analysis, we employ a data-driven approach to choose the most relevant features by evaluating their frequency in the existing literature. From this literature analysis, we identify the top two features. The primary focus of our analysis is on the feature labeled **Anxiety** specifically concerning its prevalence and impact in the context of post-COVID-19. We aim to harness machine learning algorithms to delve deeper into understanding and potentially predicting the various aspects of anxiety in individuals who have recovered from COVID-19.

### Data overview

The data overview shown in Fig. 4 for the target class amount is to gain a clear understanding of the distribution of the target class within the dataset. The amount of each target class (after anxiety) is presented in Fig. 4a. The differences of the target class amount before and after covid-19 is shown in Fig. 4b This overview helps data analysts and machine learning practitioners assess whether the dataset is balanced or imbalanced, which is crucial for making informed decisions regarding model selection, evaluation, and potential data preprocessing techniques to address class imbalances.

#### 3D visualization of data

The purpose of using t-SNE (t-Distributed Stochastic Neighbor Embedding) visualization shown in Fig. 5 is to present reduced dimensionality of complex datasets while preserving meaningful patterns and structures. It is particularly valuable for exploring and visualizing high-dimensional data in a lower-dimensional space, making it easier to identify clusters, similarities, and relationships between data points.

### Data Preprocessing

Data preprocessing is followed by survey data collection. The raw data is full of missing values and outliers. Frequently used values replace the categorical missing values. The mean value replaces numerical missing values. It is an essential step in preparing data for machine learning. Actually, it involves tasks like handling missing values, outlier treatment, scaling, encoding categorical variables, and feature selection, all of which are necessary to ensure the data is clean, standardized, and suitable for training models. Proper data preprocessing enhances model accuracy and performance^31^^32^.

### Developing the ML cassifiers

The survey dataset possesses the different demographic profiles of the people of Bangladesh. The responses are basically about how they experience certain physiological & neurological factors before & after suffering from COVID-19. So, taking the demographic profile parameters & before the experience of a factor as the independent variable & after the experience of that particular factor as the target variable, Machine Learning algorithms can predict the level of after expertise. In this research, two types of ML models are used to predict the level of health factors: Traditional ML models and Ensemble ML models, because of the use of these models in previous research articles^33^. The generalization capacity of an ensemble, which comprises numerous learners, is significantly stronger than that of individual weak learners^34^. Some of the traditional ML models are Random Forest, Decision Tree, etc. Ensemble ML models have been used in the prediction like Adaboost, Gradient Boosting, etc. our methodological implementation is presented in Algorithm 1. The outlined process in the provided text represents a comprehensive workflow for the analysis of post-COVID anxiety effects using machine learning and data analysis techniques. It begins with the reprocessing of data, including handling null values, type conversion, and normalization to ensure the dataset’s quality and consistency. The subsequent steps involve feature analysis through methods like Ordinary Least Squares (OLS), Chi-Square, and Pearson Correlation to identify the most relevant variables. The dataset is then split into training and testing portions, with 70% allocated for training the machine learning models. Four different models, including Random Forest, Decision Tree, AdaBoost, and Gradient Boost, are utilized, each with a set of parameters for evaluation. Performance metrics, including accuracy, precision, recall, and F1 score, are calculated to assess the models’ effectiveness. Confusion metrics and ROC curves are generated for a deeper understanding of model performance. Feature importance is analyzed using multiple methods, and the most influential features for post-COVID anxiety effects are recommended based on the SHAP Value analysis of the model with the highest accuracy. This workflow represents a systematic and data-driven approach to understanding the impact of post-COVID anxiety on individuals. Regenerate

During training, the Decision Tree classifier got a higher ROC curve area of 0.95. On the other hand, Adaboost classifier gains a lower ROC curve area of 0.68. We also present individual class performance with ROC curve area analysis in Fig. 9.

The description of the development of a total 4 ML classifiers is discussed in detail:

#### AdaBoost

A well-known ensemble learning technique called AdaBoost combines weak learners to produce strong learners. Each weak learner in AdaBoost receives training on some of the training data and is given a weight depending on accuracy. A weighted mixture of the poor learners–with more weights given to the more accurate ones–makes up the final model.

Here, all training examples are given identical weights in the AdaBoost model, which is generated with default hyperparameters of a maximum depth of 1 for weak learners and 50 estimators. In succeeding weak learners, the weights of misclassified instances are incrementally increased.

#### GradientBoost

A strong predictive model is produced using the effective machine learning technique gradient boost by combining many weak models. It is a specific kind of ensemble learning method that functions by repeatedly adding new models to the ensemble while repairing the flaws in the prior models. GradientBoost is especially useful for regression and classification issues with huge datasets and high-dimensional feature spaces since it use gradient descent to optimize the loss function.

Here, the gradient boost model is built with default hyperparameters such as decision trees as the base estimator, a learning rate of 0.1, a maximum depth of 3 for the trees, and 100 estimators.

#### Decision tree

A well-liked supervised learning approach for both classification and regression analysis is decision trees. The method works by dividing the feature space into subsets according to the values of the input characteristics, resulting in a model that resembles a tree and is simple for people to understand. Each leaf node of the tree represents a projected output value, and each internal node reflects a choice based on a feature value.

Here, the decision tree model is built with default hyperparameters such as the Gini impurity criterion for measuring the quality of splits, no limits on the maximum depth or number of leaf nodes, and no constraints on the minimum number of samples required to split an internal node or form a leaf node.

#### Random forest

Random Forest is an ensemble learning algorithm that builds multiple decision trees and combines them to produce more accurate predictions. Each decision tree is trained on a randomly selected subset of the training data and a random subset of the input features, ensuring diversity among the trees. The final prediction is made by averaging the predictions of all the individual trees, resulting in a more robust and accurate model that is less prone to overfitting than a single decision tree.

Here, the random forest model is built with default hyperparameters such as the Gini impurity criterion for measuring the quality of splits, several decision trees equal to 100, and a maximum depth of each tree equal to None (unlimited).

Overall parameter tuning for each model is presented in the Table 4.

**Table 4:** Model parameter selection.

Number of samples	Number of features	Performance metrics	Number information	Number of redundant	Random state	Shuffle
1000	Thirteen	MAE, MSE, Accuracy, r2 value, Precession, Recall, F1 Score	Two	Zero	Zero	False

## Result analysis: ML models to predict post-COVID-19 health factors

The data is split and we used 70% data for training and took 521 samples for testing. All the models are trained on training data. In training the model, a default hyperparameter is used. Then the models were tested on test data subsequently. Lastly, a comparison is made among the ML models test prediction which depicts a picture of a more accurate ML model for specific factors after the experience.

In Table 5, we depict the four best predictive models used for testing across various factors, along with the evaluation of their performance parameters. These parameters encompass Confusion Metrics such as Accuracy, Precision, Recall, Mean Absolute Error (MAE), Mean Squared Error (MSE), Root Mean Squared Error (RMSE), R-squared (r2), and F1 Score.

**Table 5:** Model Overall performance.

Model name	Accuracy	MAE	MSE	RMSE	R2 value
Model performance (Overall) during training					
Adaboost	0.47	1.07	2.58	1.60	–0.66
Gradient Boost	0.93	0.18	0.55	0.74	0.64
Decision Tree	0.93	0.18	0.55	0.74	0.64
Random Forest	0.60	0.88	2.18	1.47	-0.47
Model performance(Overall) during testing					
Adaboost	0.49	1.00	2.35	1.53	-0.68
Gradient Boost	0.92	0.21	0.65	0.81	0.53
Decision Tree	0.92	0.21	0.65	0.81	0.53
Random Forest	0.59	0.90	2.24	1.49	-0.528

Table 6 provides a detailed overview of the training performance for each target class. In Table 7, we present a corresponding breakdown of the testing performance for each target class.

**Table 6:** Training performance (Class wise).

Model	Class name	Precession	Recall	F1 Score	Support
Adaboost	Agree	1.00	1.00	1.00	204
	Neutral	0.53	0.76	0.62	261
	Disagree	0.00	0.00	0.00	58
	Strongly agree	0.29	0.16	0.21	85
	Strongly dis-agree	0.18	0.14	0.16	92
Gradient Boost	Agree	1.00	1.00	1.00	204
	Neutral	0.95	0.93	0.94	261
	Disagree	1.00	1.00	1.00	58
	Strongly agree	1.00	1.00	1.00	85
	Strongly dis-agree	0.81	0.86	0.83	92
Decision Tree	Agree	1.00	1.00	1.00	204
	Neutral	0.95	0.93	0.94	261
	Disagree	1.00	1.00	1.00	58
	Strongly agree	1.00	1.00	1.00	85
	Strongly dis-agree	0.81	0.86	0.83	92
Random Forest	Agree	1.00	1.00	1.00	204
	Neutral	0.57	1.00	0.72	261
	Disagree	1.00	0.45	0.62	58
	Strongly agree	0.00	0.00	0.00	85
	Strongly dis-agree	0.00	0.00	0.00	92

**Table 7:** Testing performance (Class wise).

Model	Class name	Precession	Recall	F1 Score	Support
Adaboost	Agree	1.00	1.00	1.00	17
	Neutral	0.54	0.77	0.63	265
	Disagree	0.00	0.00	0.00	74
	Strongly agree	0.39	0.20	0.26	86
	Strongly dis-agree	0.22	0.23	0.22	79
Gradient Boost	Agree	1.00	1.00	1.00	17
	Neutral	0.93	0.92	0.93	265
	Disagree	1.00	1.00	1.00	74
	Strongly agree	1.00	1.00	1.00	86
	Strongly dis-agree	0.75	0.77	0.76	79
Decision Tree	Agree	1.00	1.00	1.00	17
	Neutral	0.93	0.92	0.93	265
	Disagree	1.00	1.00	1.00	74
	Strongly agree	1.00	1.00	1.00	86
	Strongly dis-agree	0.75	0.77	0.76	79
Random Forest	Agree	1.00	1.00	1.00	17
	Neutral	0.55	1.00	0.71	265
	Disagree	1.00	0.50	0.67	74
	Strongly agree	0.00	0.00	0.00	86
	Strongly dis-agree	0.00	0.00	0.00	79

### Performance analysis

#### Training performance analysis

In order to thoroughly evaluate the performance of our machine learning method, we allocated a significant portion, namely 70%, of the available data for testing. Among the algorithms we employed, the Decision Tree model stood out as the top performer, boasting an impressive accuracy metric result of 93.84%. In contrast, the Gradient Boost and Ada Boost algorithms exhibited slightly lower accuracy scores when compared to the Decision Tree. To provide a comprehensive understanding of the model’s performance, we additionally reported key metrics such as Mean Absolute Error (MAE), Mean Squared Error (MSE), and the R-squared (r2) score, shedding light on aspects beyond simple accuracy. Furthermore, to gain a deeper insight into the model’s classification capabilities, we presented the results of the Confusion Matrix, offering a more granular perspective on its training performance. For a visual summary of the overall performance, please refer to the Overall training performance is shown in Table 5. And performance for each class is shown in Table 6.

#### Testing performance analysis

In order to assess the machine learning method’s performance, we reserved 30% of the data for evaluation purposes. Based on the result as shown in Tables 5 and 6, we can say that the Decision Tree model outperformed the others, achieving an impressive accuracy score of 92.70%. In contrast, both the Gradient Boost and Ada Boost algorithms exhibited slightly lower accuracy scores when compared. To provide a more comprehensive evaluation, we also reported metrics such as Mean Absolute Error (MAE), Mean Squared Error (MSE), and the R-squared (r2) score. Furthermore, we delved deeper into the testing performance by presenting the results of the Confusion Matrix, offering a more detailed insight into the model’s classification performance. Overall testing performance is shown in Table 5. And performance for each class is shown in Table 7.

#### Performance analysis using confusion matrix

**Figure 6:**
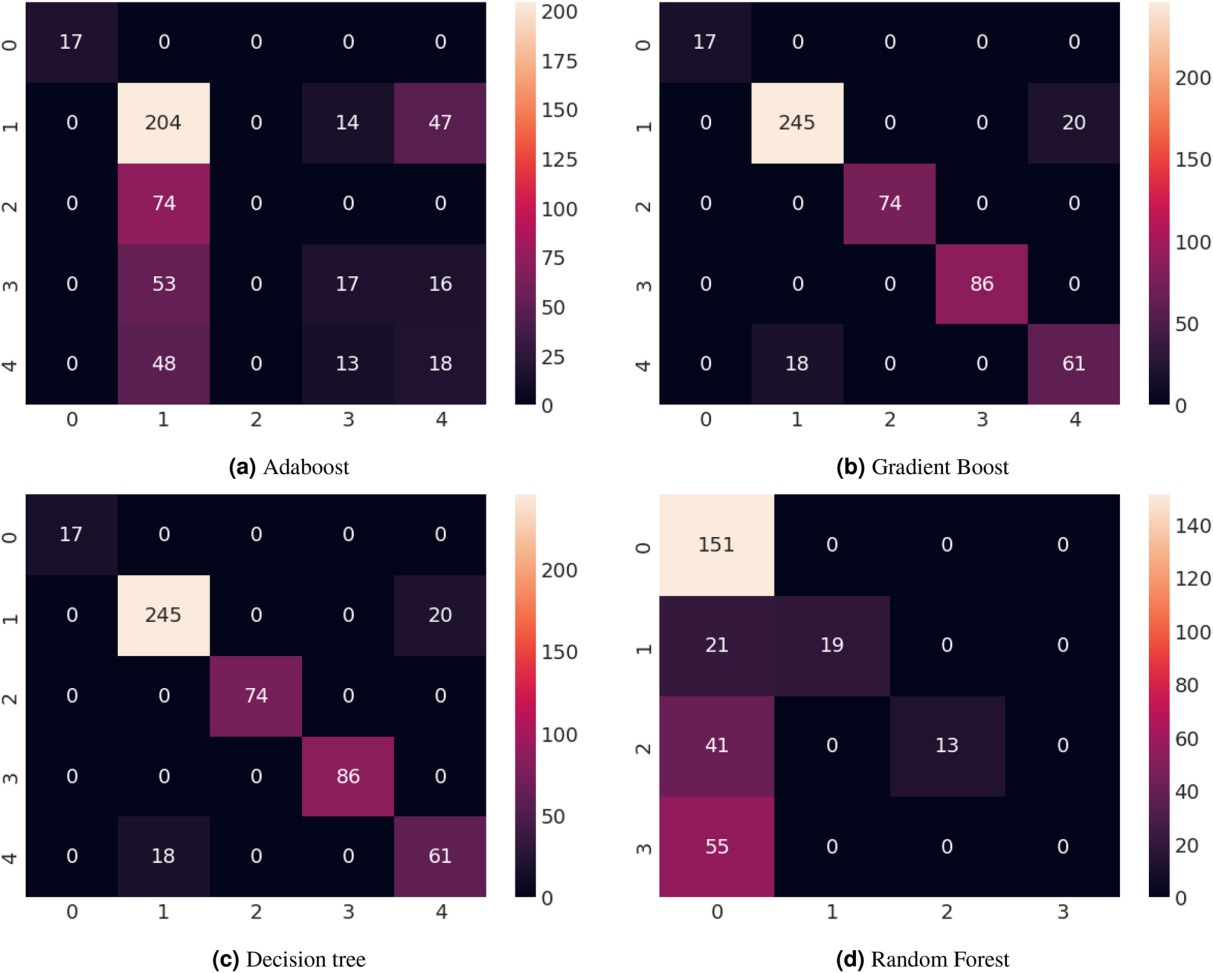
Confusion matrix testing performance for anxiety after Covid-19.

A confusion matrix is a vital tool in machine learning, especially for classification tasks. It’s a matrix summarizing how well a classification algorithm performs, giving insights into its accuracy in predicting true data classes. Rows represent actual classes, while columns represent the model’s predictions. This tool is crucial for evaluating model performance by calculating key metrics like accuracy, precision, recall, and F1-score. These metrics offer a deeper understanding of the model’s strengths and weaknesses, facilitating model refinement and enhancement. Its significance is paramount in evaluating machine learning effectiveness, especially in scenarios involving imbalanced classes or specific error types. In Fig. 6, The testing performance of four selected models is shown using the Confusion Matrix. Based on the Confusion Matrix result we can conclude that the Decision tree model performs better than all other models.

### Computational time analysis

The Table 8 displays the computational time required for training and testing various models. It demonstrates the time complexity associated with each model. According to the data, the decision tree model requires slightly less time compared to the other models. Decision trees are simpler than random forests. Decision trees consolidate decisions, while random forests combine multiple decision trees. Random forests are slower but more comprehensive, whereas decision trees are fast and efficient.

**Table 8:** Time complexity analysis.

Model	Training time (sec)	Testing time (sec)
Random forest	1.065118	0.024023
AdaBoost	1.031105	0.023526
Gradient boost	1.033102	0.023728
Decision tree	1.001170	0.021021

#### ROC curve analysis

**Figure 7:**
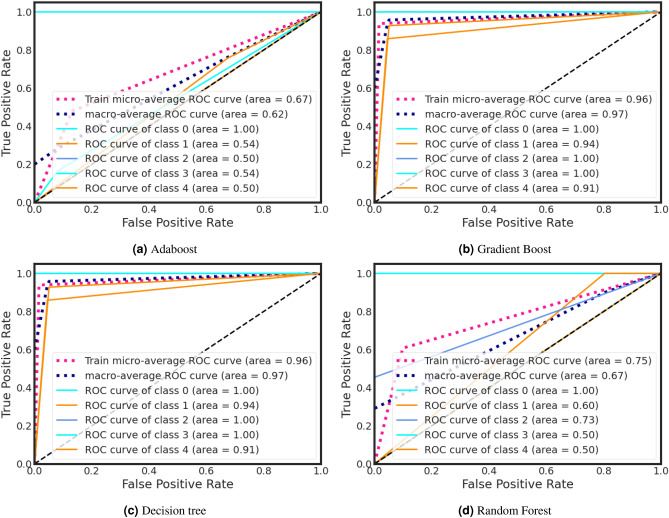
Training ROC curve for anxiety after Covid-19.

**Figure 8:**
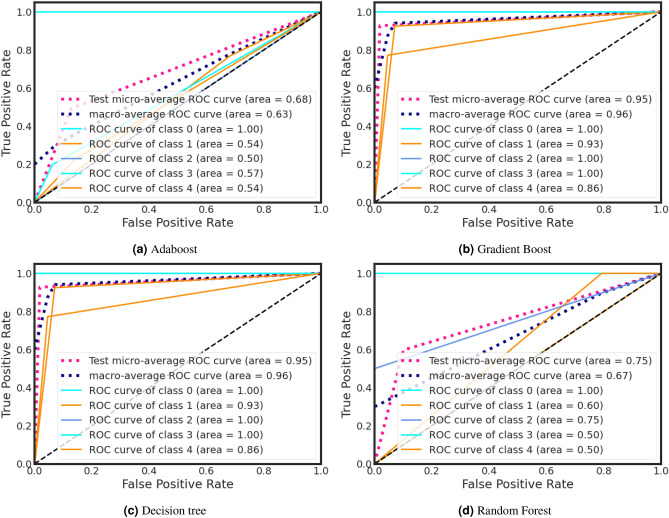
Testing ROC curve for anxiety after Covid-19.

ROC curves shown in Figs. 7 and 8 provide a powerful and intuitive means to assess binary or categorical classification model performance. This ROC curve offers a visually interpretable representation of a model’s ability to discriminate between positive and negative cases, facilitating easy model comparison and selection. ROC curves are robust to class imbalance and varying class prior probabilities, offering insights even in challenging dataset scenarios. The Area Under the ROC Curve (AUC) condenses overall model performance into a single scalar metric, simplifying model evaluation and ranking. Moreover, ROC curves are applicable to a wide range of classification algorithms, aiding transparency, interpretability, and informed decision-making, especially in fields like medicine and diagnostics where sensitivity and specificity trade-offs are critical.

During the testing phase, the Decision Tree classifier achieved a higher ROC curve area of 0.95, while the Adaboost classifier attained a lower ROC curve area of 0.68 (Fig.8). Additionally, we provide an analysis of individual class performance using ROC curve area metrics in Fig. 8.

### Important feature analysis of best models

**Figure 9:**
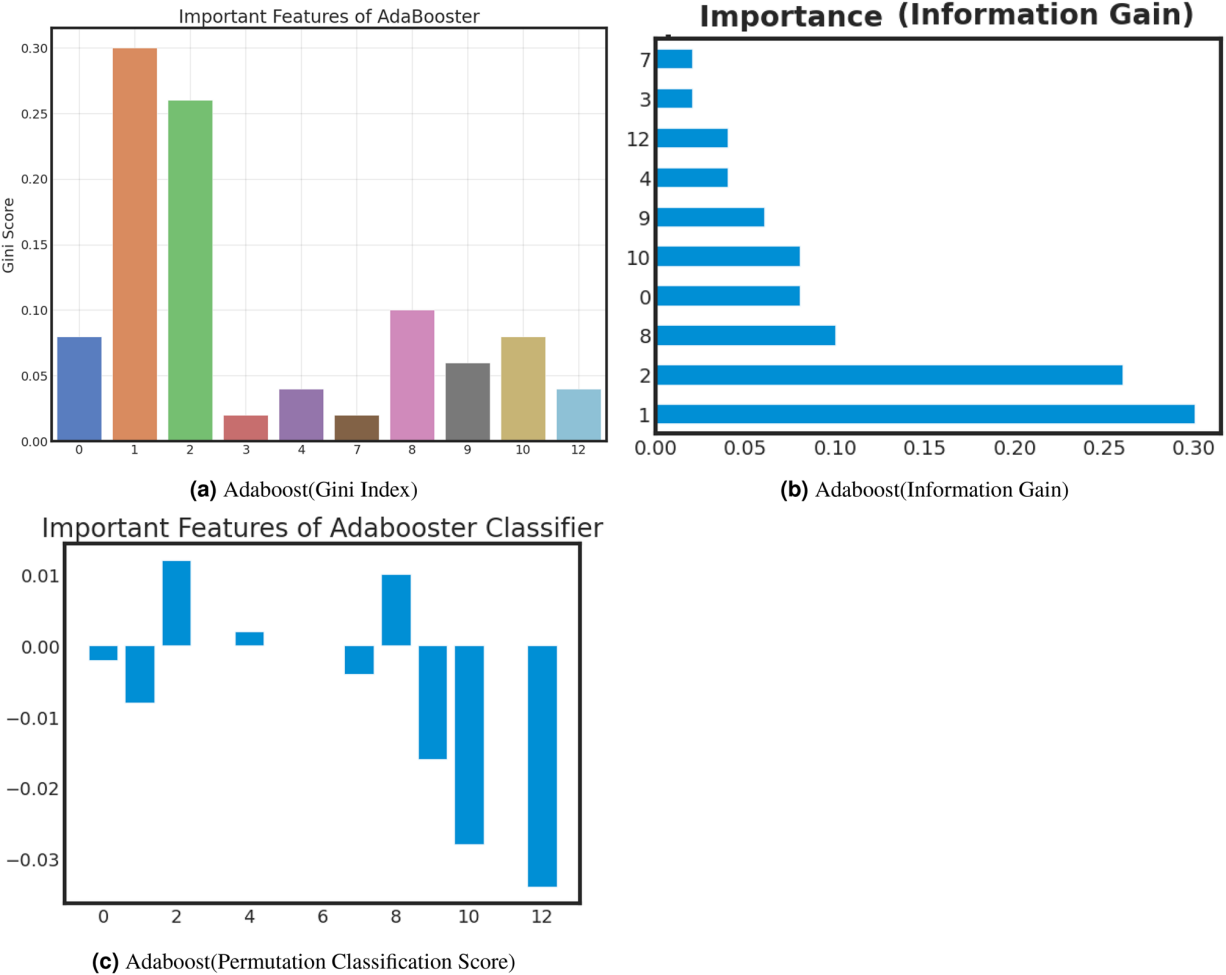
AdaBoost important feature analysis for anxiety after Covid-19.

**Figure 10:**
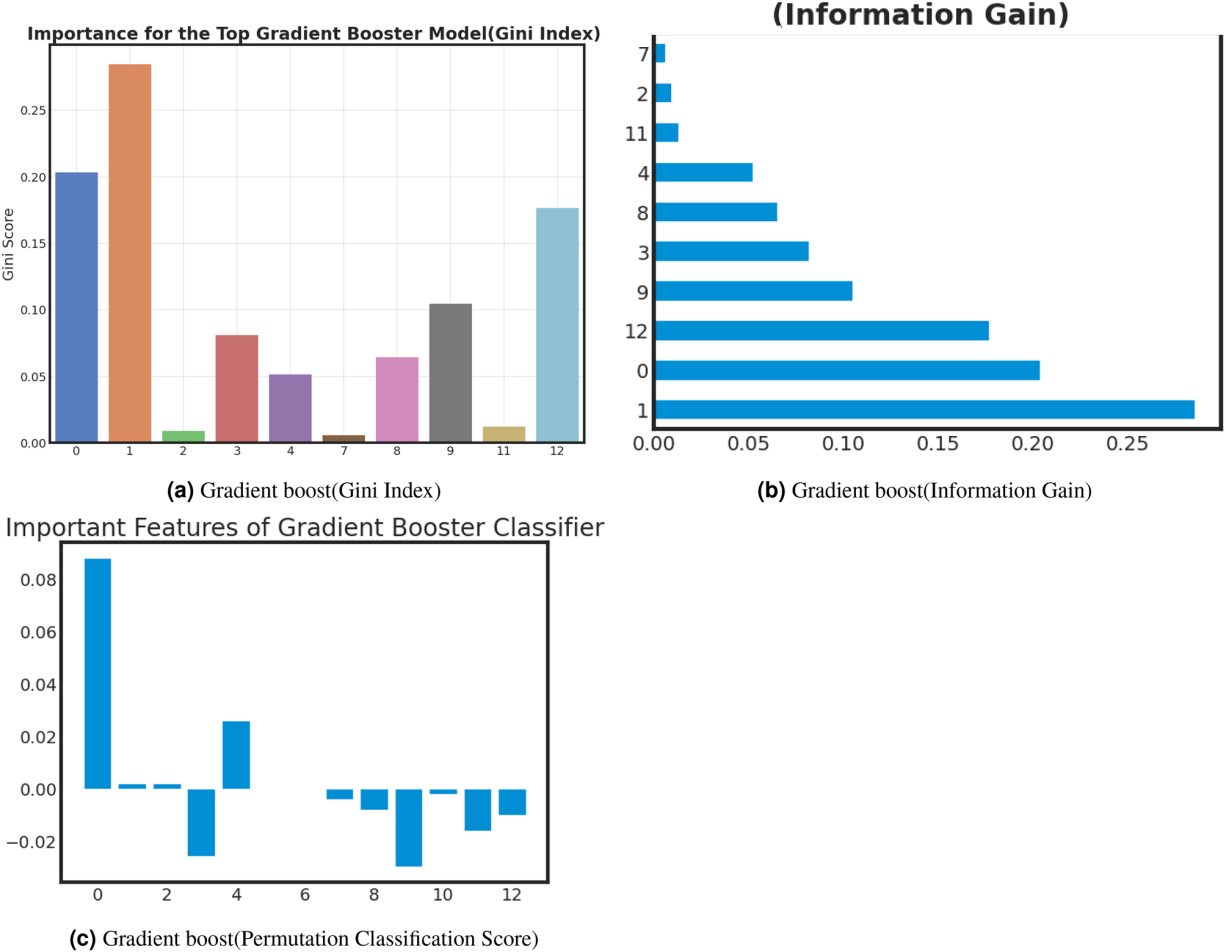
Gradient boost important feature analysis for anxiety after Covid-19.

**Figure 11:**
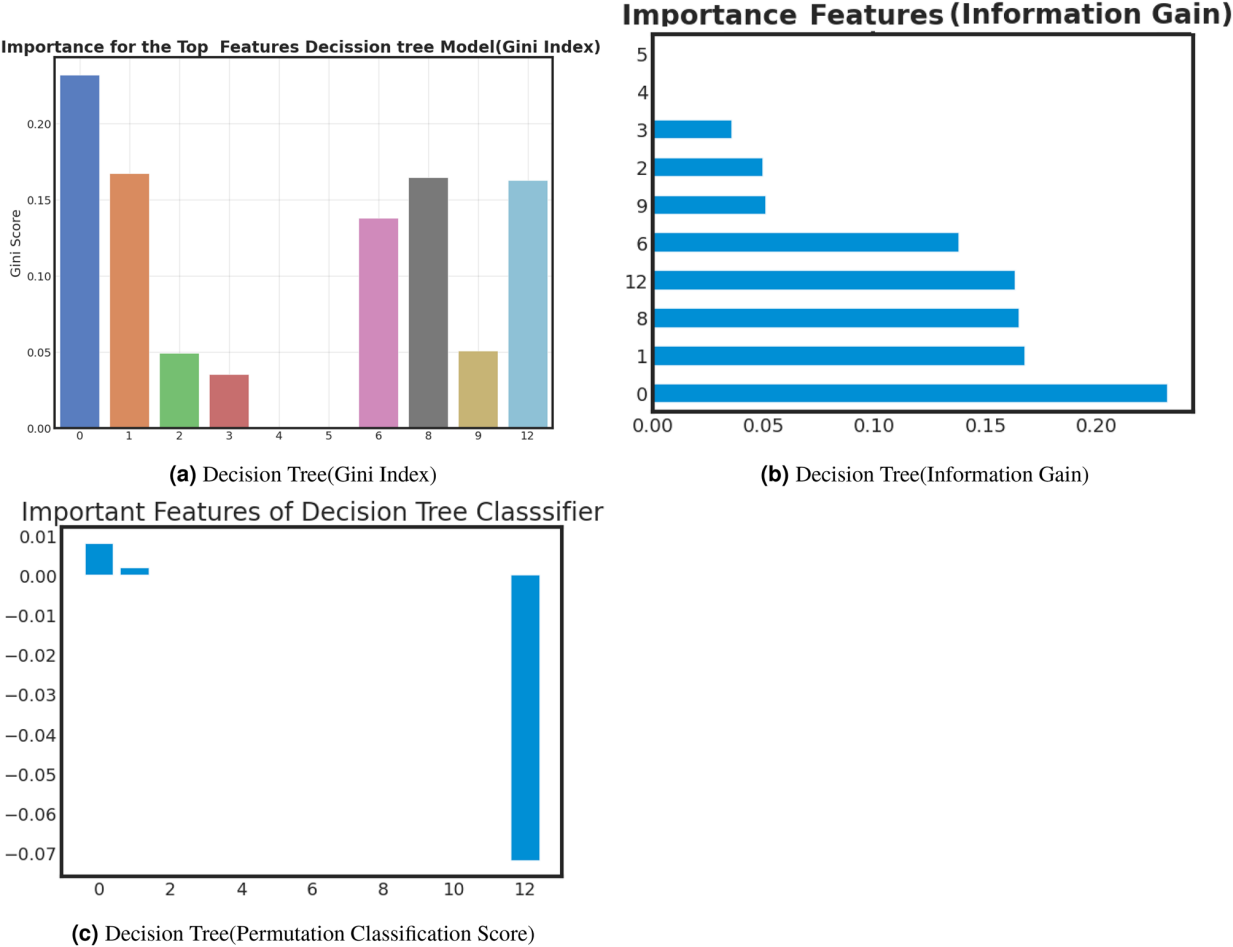
Decision tree important feature analysis for anxiety after Covid-19.

Examining critical facets within machine learning offers several benefits, including enhanced model comprehension, increased efficiency through feature selection, improved performance, data-driven decision-making, optimized resource allocation, pattern recognition, and adherence to regulatory requirements. Furthermore, this analysis validates expertise in the relevant field, identifies potential biases, aids in model explanation, and fosters adaptability to changing circumstances. we analyze important features of best-performed model with Gini Index, Information Gain, and Classification Permutation. Figure 9, 10 and 11 present the exploration of important factors anxiety for the post-COVID-19 effect.

**Figure 12:**
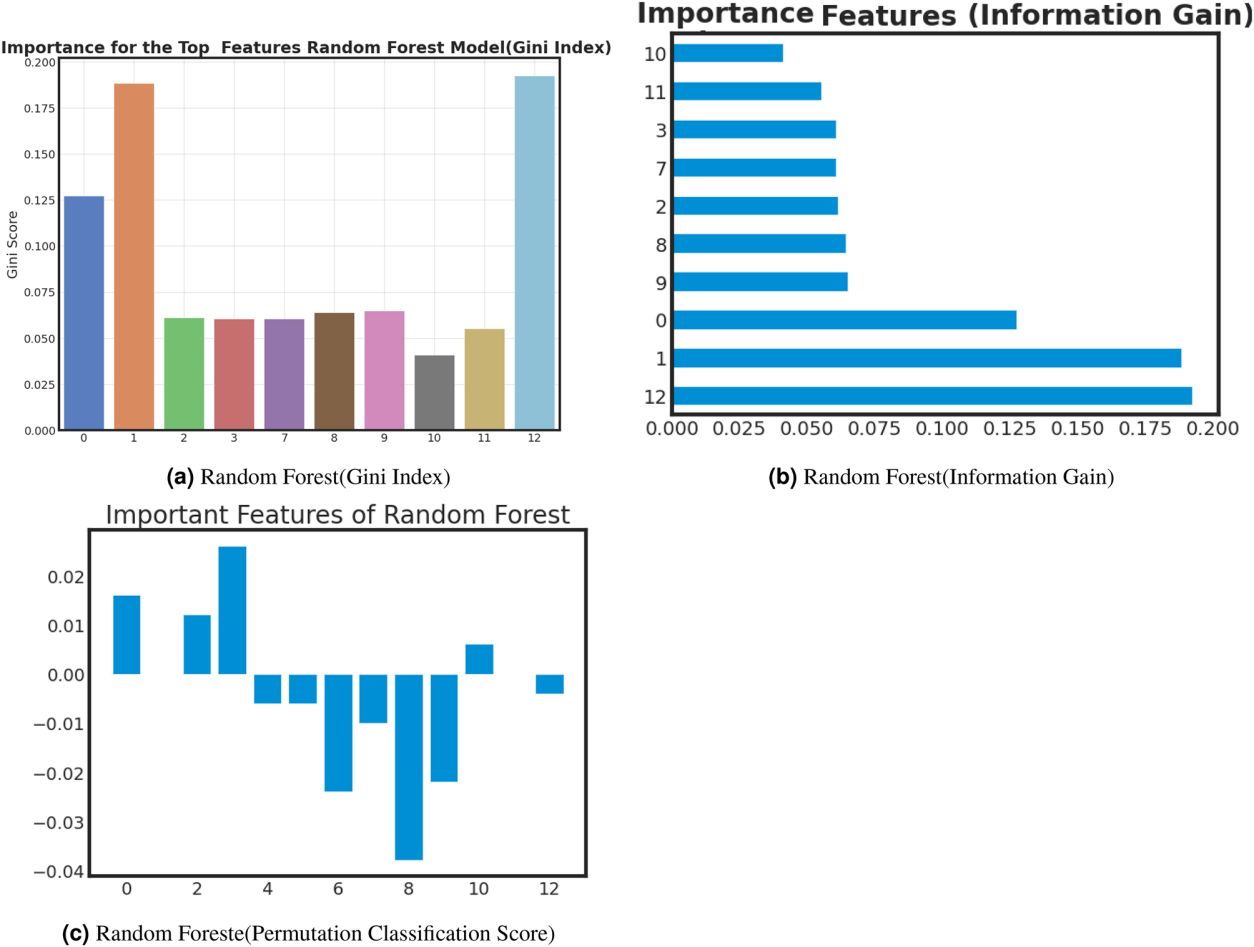
Random forest important feature analysis for Anxiety After Covid-19.

**Figure 13:**
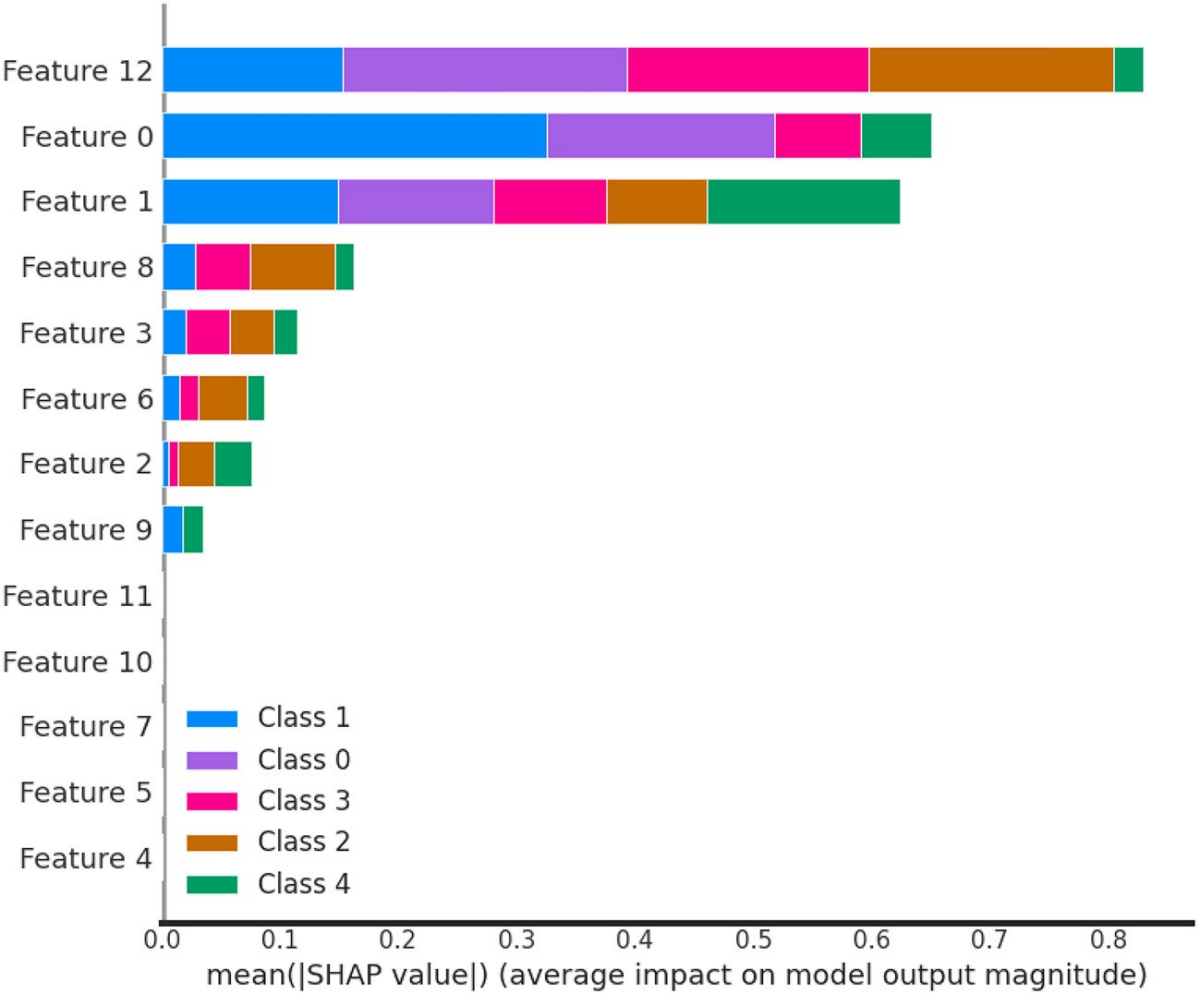
SHAP value analysis for decision tree algorithm.

1. *Important feature exploration using Gini Index for the best model*: The Gini Index, often used in decision tree algorithms, serves the purpose of quantifying the impurity or disorder within a set of data points within a specific class. It provides a measure of how frequently a randomly chosen element would be misclassified in terms of its class label if it were randomly assigned a label based on the distribution of class labels in the data subset. In the context of decision trees, the Gini Index is employed as a criterion for selecting the best feature to split data on, aiming to minimize this impurity. A lower Gini Index indicates a purer node with data points predominantly belonging to a single class, making it a valuable tool for guiding the creation of decision tree nodes that effectively partition data into more homogenous subsets, leading to better classification performance.
  Based on the Gini analysis as shown in Figs. 10a, 11a, 12a and 13a, we clearly show that the decision tree, Gradient boost, Adaboost and Random forest algorithm give the most priority to features 0, 1 and 12.
2. *Important feature exploration using information Gain for the best model*: The purpose of Information Gain in the context of decision trees and feature selection is to quantify how much knowledge or reduction in uncertainty a particular feature provides when used to split a dataset. It measures the difference in entropy (or impurity) between the original dataset and the subsets created by splitting the data based on that feature. By selecting the feature with the highest Information Gain, decision tree algorithms aim to identify the feature that can separate the data into more homogenous or pure subsets, leading to more effective and accurate classification or regression models. Essentially, Information Gain helps decision trees make informed choices about which features to use as decision criteria, facilitating the creation of a tree structure that best represents the underlying data patterns.
  Based on the Information gain analysis, Figs. 10b, 11b, 12b and 13b, we clearly show that the decision tree, Gradient boost, Adaboost and Random forest algorithm give the most priority to features 0, 1 and 12.
3. *Important feature exploration using classification permutation for the best model*: The purpose of Feature Importance by classification permutation is to assess the relative significance of individual features in a machine-learning classification model. It achieves this by systematically shuffling the values of a single feature while keeping all other features constant and then measuring the resulting drop in the model’s performance metric (typically accuracy or F1 score). Features that, when shuffled, cause a significant decrease in model performance are considered important, as they carry valuable information for making accurate predictions. This method helps practitioners identify which features contribute most to the model’s predictive power, aiding in feature selection, model interpretation, and improving overall model performance by focusing on the most informative attributes.
  Based on the Permutation feature analysis, Figs. 10c, 11c, 12c and 13c, we clearly show that the Decision tree, Gradient boost, Adaboost and Random forest algorithm give the most priority to features 0 and 1.

Noted that, all covid patient Features are labeled as 0: Gender, 1: Age and 12: Vaccination Status.

#### Important feature analysis of trained models based on SHAP value

SHAP (SHapley Additive exPlanations) value analysis shown in Fig. 13 offers several notable advantages in the realm of model interpretability and feature analysis. One key advantage is its ability to provide a clear and intuitive understanding of how individual features influence the predictions of machine learning models. By assigning importance scores to each feature, SHAP values allow data scientists and stakeholders to pinpoint the most influential factors behind model outcomes, facilitating informed decision-making and actionable insights.

Furthermore, SHAP values ensure consistency in attribution, meaning that the sum of SHAP values for all features equals the difference between the model’s prediction and the expected (average) prediction. This consistency lends credibility to the interpretability of the analysis and ensures that the contributions of each feature align with the overall prediction.

Moreover, SHAP values offer interpretability across a wide range of machine learning models, including complex algorithms like gradient boosting and deep neural networks. This versatility makes SHAP a valuable tool in various domains, from healthcare to finance, where model transparency and trust are paramount.

Finally, SHAP values can be visualized in multiple ways, including summary plots, force plots, and dependence plots, making it accessible for both technical and non-technical stakeholders. These visualizations enhance the communication of model insights and contribute to more effective collaboration between data scientists and domain experts. In summary, SHAP value analysis significantly advances the field of model interpretability by offering transparency, consistency, and versatility in understanding the driving forces behind machine learning predictions.

Based on the SHAP value analysis of the Decision Tree algorithm, feature 12, feature 0 and feature 1 are the most important features. All covid patient Features are labelling as 0: Gender, 1: Age and 12: Vaccination Status.

## Comparative analysis

In the comparative analysis, we compared our model with state-of-the-art. We compared our method with some relevant existing methods. Based on the Table 9, our method obtains higher accuracy and handles more target classes.

**Table 9:** Comparative analysis.

Refs.	Method	Findings	Total features: Input features	Number of class: Target class	Number of sample	Performance
^35^	Stacking ensemble	Finds heart disease of the post covid patient	11: age, gender, weight, height, smoking status, pregnancy status, diabetes or other diseases, and vaccination	6: shortness of breath, fever, cough, fatigue, dizziness, loss of taste and smell	180	Accuracy: 93%, Specificity: 95% Precision: 95%, and recall :92.05%
^36^	Decision tree	Analyzing COVID-19 vaccine side effects: machine learning & statistics.	86: age, gender, comorbidity history, allergic history, and birth defect information after vaccination, vaccination date, date of reaction onset, hospitalization information	2: complication-free vaccination or not	72,147	Accuracy: 90%
^37^	Hybrid ensemble	Presenting a hybrid ensemble machine learning model for assessing severity risk and predicting post-COVID outcomes.	35: age, weight, height, BMI, CAT, pulse, Physiological and biomarker’s features	2: Severity risk or not	122	Accuracy: 92.4%
^38^	XGB	Detecting modifiable predictors of COVID-19 vaccine side effects using machine learning techniques.	NA: Medical and demographic features	2: Alergic and non algergic	50484	Accuracy: 84%
Ours	Decision tree	Examining post-pandemic health: a thorough exploration of post-COVID-19 health impacts and feature analysis utilizing advanced machine learning models.	13: “gender”, “age”, “edu- cation”, “heartd isease”, “diabetis”, “otherd isease”, “smoking”, “bloodpressure”, “weight”, “workt ype” , “married”, “vaccinationstatus”, “vaccinationd osestatus”.	5: Anxiety: Strongly agree, Agree, Neutral, Disagree, and Strongly disagree	1000	Accuracy: 93.84% MAE: 0.18, MSE: 0.55, RMSE: 0.74 and r2 value: -0.66

### **Novely of our research**

Our research brings the following contributions to the field:

1. *Comprehensive health factor analysis*: One of the main contributions of our research is the comprehensive analysis of 17 significant health factors associated with COVID-19. These factors encompass both Physiological and Neurological aspects, providing a holistic view of the health complications linked to the disease. This extensive factor analysis is crucial in understanding the multifaceted impact of COVID-19 on individuals’ health.
2. *Longitudinal surveys for pre-and post-illness assessment*: We have undertaken a distinctive approach by conducting surveys with individuals who have recovered from COVID-19. These surveys assess their health conditions both before and after the illness, creating a longitudinal perspective. This approach enables us to track the progression of health complications, which is a novel aspect of our research.
3. *Rigorous statistical analysis*: Our research stands out for its rigorous statistical analysis of the survey data. By subjecting the data to an in-depth statistical examination, we unveil how each of the 17 health factors independently influences patients. This analytical rigor provides a deeper understanding of the individual and collective impact of these factors.
4. *Effective predictive models*: Our study identifies the Decision Tree algorithm as the most effective predictive model for evaluating the influence of health factors, specifically in predicting anxiety levels. This contribution enhances the precision and reliability of post-COVID-19 health assessments.
5. *Innovative feature analysis methods*: In the final stage of our research, we employ a variety of innovative methods to identify key features in the post-effects of the best-performing machine learning model. These methods include feature importance analysis, Gini index, information gain, feature importance permutation, and SHAP value analysis. This feature analysis approach adds a novel dimension to the understanding of post-COVID-19 health outcomes.

### **Implication of the research**

The implications of our research extend to following areas that offer valuable insights and potential benefits for healthcare, public health, and research endeavors:

1. *Improved post-COVID-19 patient care*: Our research provides a deeper understanding of the health complications associated with COVID-19, allowing healthcare providers to offer more tailored and effective care to individuals recovering from the disease. Identifying the key health factors and their impact can aid in personalized treatment plans.
2. *Early intervention and monitoring*: The longitudinal approach in our study allows for the early identification of health complications that may arise post-COVID-19. This early detection can lead to timely interventions and monitoring to prevent or mitigate the severity of these complications.
3. *Resource allocation*: Health systems can use our findings to allocate resources more effectively. By understanding the specific health factors that influence patients, healthcare facilities can allocate resources to address the most pressing needs, optimizing patient care.
4. *Public health planning*: Public health authorities can benefit from our research in planning and implementing post-COVID-19 health strategies. Understanding the factors contributing to health complications can inform public health policies and interventions to support affected individuals.
5. *Research advancements*: Our research contributes to the growing body of knowledge about the long-term effects of COVID-19. It provides a basis for further research and investigations into the intricacies of post-COVID-19 health, fostering a better understanding of this emerging field.
6. *Machine learning applications*: The effectiveness of the Decision Tree algorithm in predicting anxiety levels and the innovative feature analysis methods can inspire future machine learning applications in healthcare and predictive modeling.

## Conclusion

Our research focused on analyzing major health complications related to COVID-19, identifying 17 significant health factors categorized as Physiological and Neurological. We conducted surveys with recovered COVID-19 patients to assess the impact of these factors on their health before and after their illness. We rigorously analyzed the survey data, examining the independent influence of each factor and their interconnections. We chose the most important feature named Anxiety from the outcome of the survey study frequency. Among four ML models, the Decision Tree algorithm demonstrated the highest accuracy in predicting anxiety levels, which was our primary objective. Finally, we identified key features in the post-effects of the best-performing machine learning model through various methods, providing valuable insights into post COVID-19 effects.

Post-COVID traumas have both mental and physical effects, significantly impacting patients’ lives. Depression doubled from 20% to 37%, while vigilance dropped from 60% to 16.67%, impulsiveness decreased from 33.33% to 20%, and determination fell from 60% to 20%. Confidence levels plummeted from 56.67% to 20%, and energy levels declined from 56.67% to 16.67%. Relationships exist among factors like chest pain and unhappiness, sleep and attentiveness, with forgetfulness having connections with almost all other factors. Additionally, there are direct or inverse relationships among various factors, with depression and forgetfulness showing a direct relationship (p-value = 0.678515), and anxiety and energy displaying an inverse relationship (p-value = -0.18056).

This signifies that a COVID survivor suffering more anxiety will most probably feel less energetic. Lastly, we discovered the best predictive ML models to predict the degree of impact on post-COVID-19 health factors. It is observed that our developed Decision Tree model showed the highest accuracy(0.9384) to predict the degree of impact in case of Anxiety in a post-COVID individual. Similarly, developed Decision Tree models were also identified as the most accurate model in predicting the degree of impact in case of Anxiety. In summary, different predictive machine learning models showed a definite accuracy in predicting the degree of impact of various factors in post-COVID-19 individuals.

## Data Availability

The datasets generated and analyzed during the current study are publicly available in our GitHub repository link at https://github.com/shafiq-islam-cse/Data---Exploring-Post-COVID-19-Health-Effects-and-Features-with-Advanced-Machine-Learning-Techniques.
